# Raman Spectra and Ancient Life: Vibrational ID Profiles of Fossilized (Bone) Tissues

**DOI:** 10.3390/ijms231810689

**Published:** 2022-09-14

**Authors:** Zuzana Jurašeková, Gabriela Fabriciová, Luis F. Silveira, Yuong-Nam Lee, Jaroslav M. Gutak, Majid Mirzaie Ataabadi, Martin Kundrát

**Affiliations:** 1 Department of Biophysics, Faculty of Science, Pavol Jozef Šafárik University in Košice, Jesenná 5, 04154 Košice, Slovakia; 2 Museu de Zoologia da Universidade de São Paulo, Caixa Postal 42.494, São Paulo 04218-970, Brazil; 3 School of Earth and Environmental Sciences, Seoul National University, Seoul 08826, Korea; 4 Department of Geology, Geodesy, and Life Security, Institute of Mining and Geosystems, Siberian State Industrial University, Kirov Street 42, 654007 Novokuznetsk, Russia; 5 Department of Geology, Faculty of Science, University of Zanjan, Zanjan 4537138791, Iran; 6 PaleoBioImaging Lab, Evolutionary Biodiversity Research Group, Center for Interdisciplinary Biosciences, Technology and Innovation Park, Pavol Jozef Šafárik University in Košice, Jesenná 5, 04154 Košice, Slovakia

**Keywords:** micro-Raman spectroscopy and imaging, spectral markers, fossilized tissue, bone, diagenesis

## Abstract

Raman micro-spectroscopy is a non-destructive and non-contact analytical technique that combines microscopy and spectroscopy, thus providing a potential for non-invasive and in situ molecular identification, even over heterogeneous and rare samples such as fossilized tissues. Recently, chemical imaging techniques have become an increasingly popular tool for characterizing trace elements, isotopic information, and organic markers in fossils. Raman spectroscopy also shows a growing potential in understanding bone microstructure, chemical composition, and mineral assemblance affected by diagenetic processes. In our lab, we have investigated a wide range of different fossil tissues, mainly of Mesozoic vertebrates (from Jurassic through Cretaceous). Besides standard spectra of sedimentary rocks, including pigment contamination, our Raman spectra also exhibit interesting spectral features in the 1200–1800 cm^−1^ spectral range, where Raman bands of proteins, nucleic acids, and other organic molecules can be identified. In the present study, we discuss both a possible origin of the observed bands of ancient organic residues and difficulties with definition of the specific spectral markers in fossilized soft and hard tissues.

## 1. Introduction

Fossilized hard tissues are the most common remains of extinct vertebrates. In fact, bones and teeth are mainly the only surviving materials available for scientific analysis. They represent archives that can be used to reconstruct the past life of ancient populations and paleoenvironmental conditions. While buried, bones undergo several taphonomic stages and diagenetic transformations, the intensity of which depends upon a variety of complex processes and geochemical factors that cause the postmortem alteration of their microstructure and composition [1,2,3,4]. Over the past few decades, the survival of biomolecules in archaeological artefacts and fossil bones has become one of the most debated research areas [5,6,7,8,9,10,11,12,13,14]. Nevertheless, understanding the structural complexity of mineralized tissues and the fossilization process itself remains a crucial issue [15,16,17]. Hence, a detailed study on bone diagenesis continues to have a double purpose: (*i*) to assess the preservation state of paleorganic residues and (*ii*) to understand the alteration pathways of fossilized tissues.

Various analytical techniques (individually as well as combined in multiple-technique approaches [18,19]) are widely used in paleontology to study subfossil and fossil bones, to assess their preservation state and to understand mechanisms of diagenetic changes. In particular, these techniques include optical microscopy [20] and scanning and transmission electron microscopy [21,22,23], together with the elemental analysis (energy dispersive spectroscopy) [24], X-ray diffraction [21,25,26], Fourier-transform infrared spectroscopy (FTIR) [21,25,27,28,29], Raman spectroscopy [30,31,32,33,34], and X-ray fluorescence [22,35,36], as well as more invasive approaches, including liquid and gas chromatography coupled to tandem mass spectrometry [37,38,39].

Different analytical techniques probe different physical and chemical properties and provide information at different scale levels. Paleohistological and other microstructural analyses demonstrate variable types and extents of diagenetic alteration [40,41]. Furthermore, synchrotron-based imaging techniques are increasingly used to provide non-destructively novel 3D insights into histology of fossil tissues at the submicron scale [42,43,44].

In the case of archaeological and paleontological samples, when usually only a tiny amount of rare material is available, both the amount of sample and the preparation protocol required for the analysis are non-negligible issues that determine selection of the employed techniques. Therefore, the choice of the most appropriate procedure is based on the study’s objectives and information obtained by its use; whereas non-destructive analyses are preferred over destructive ones. However, the overall experimental costs also play a specific involvement in the possible use of a particular technique.

Diagenesis-induced chemical alterations in bone can be accurately examined by optical vibrational spectroscopy. FTIR is the most commonly used vibrational spectroscopic technique to discriminate between fossilized, subfossilized, and recent (modern) bones. It has been extensively applied in material characterization and bone diagenesis studies [16,18,21,28,45,46]. In particular, parameters such as bone crystallinity, carbonate, and organic contents can be routinely evaluated [27,28,29,47]. The postmortem interval can be estimated as well [48]. Raman spectroscopy (RS) also provides a vibrational spectrum, whereas Infrared (IR) and RS techniques give complementary information on quantity and quality of the composition of bone [49]. For instance, RS is more sensitive than IR for the inorganic matrix of the bone [50]. Moreover, RS demonstrates several advantages over IR: minimal sample preparation, higher spectral and spatial resolution, higher analytical speed, and better applicability to fresh tissues [49,51].

Raman spectroscopy is an established spectroscopic technique used to assess scattered light from heterogeneous materials. The frequency of this light depends on the structural characteristics of the molecular bonds and their chemical environments. In general, RS enables samples in any physical state to be qualitatively and sometimes also quantitatively examined, and different phases of the analyzed material can also be distinguished [52]. In fact, micro-Raman spectroscopy (or Raman micro(spectro)scopy) is an analytical technique of a high spatial resolution that enables performing selective analyses reaching the micron resolution [38]. Subsequently, Raman (multi)spectral mapping images provide detailed chemical information together with the spatial distribution. Finally, RS can be recognized as a non-invasive technique, since it does not require labeling, staining, or other forms of destructive sampling, such as extracting the target material from analyzed specimens [53].

When applied to bone material characterized by the intrinsic spatial heterogeneity of bone tissue, organic and mineral phases can be simultaneously investigated. Similarly, as in the case of IR, parameters related to the collagen and carbonates contents, their substitution, and the bioapatite crystallinity can be calculated from the recorded Raman spectra [21,32,54,55,56]. Nowadays, Raman microscopy and imaging techniques are frequently used to assess bone material characterization in the field of biomedical and biomaterial applications [30,31,32,57,58,59,60,61,62,63,64,65,66]. Besides, it is also an effective and highly informative tool for studying the diagenetic alteration of subfossil and fossil bones [21,31,34,38,53,54,55,67]. RS has been applied to evaluate changes in bone composition associated with aging, disease, and environmental conditions and to model diagenetic processes involved in bone alteration along with its localization within the sample [31,32,48,68,69]. RS also significantly helps to analyze the preservation of microfossils at a high spatial resolution, as 3D chemical and structural maps can be achieved while maintaining the chemistry and morphology of the sample intact [34]. Undoubtedly, RS is increasingly popular for investigating the preservation of biological and geological signatures in fossilized tissues and characterizing isotopic signatures, trace elements, and organic biomarkers found in fossils [52,70,71,72,73,74]. Various groups have used RS to identify the origins of compounds and structures found in fossils and to understand the patterns and processes involved in fossilization of biomolecules; the extended work of Wiemann et al. deserves special attention in this sense [9,10,75,76,77,78]. Finally, RS is also a promising screening method to investigate the bone preservation state and select the most suitable samples or their fractions to carry out further well-designed experiments [70].

Executing Raman spectroscopic analysis of bone material, one must also consider various limitations. Only the principal components of bone are usually observed spectroscopically, although non-collagenous proteins also contribute to the overall bone Raman spectrum. However, their low abundance and spectral similarity with the collagen signal make their detection difficult [32]. Bone lipids and phospholipids are not frequently detected because of their easy removal during sample treatment [79]. In contrast, an intense fluorescence signal, mainly induced by organic constituents [48,49], can partially or even completely obscure the characteristic peaks of analyzed bone sample. Finally, RS is a surface analytical technique that, especially in the case of thick, opaque, and heterogeneous samples as fossil bones are, may require other experimental approaches; for instance, coupling to a confocal microscope, SORS (Spatially-offset Raman spectroscopy), or even additional sample treatment (e.g., preparation of thin slices) [48,72].

This study aims to demonstrate the effectiveness and analytical potential of Raman microspectroscopy for studying the preservation state of subfossil and fossil bones and their qualitative characterization. In addition, we discuss here putative origins of intense spectral features recorded in the analyzed fossil bone samples. Although these bands are visible in the spectral region characteristic of the biomolecules (above 1000 cm^−1^) [80,81,82], their detection is only rarely reported in literature [52,83,84]. We formulate their possible definitions as specific spectral markers for fossilized tissues. Simultaneously, we present a brief overview of using Raman spectroscopy in molecular paleobiology, particularly in the fossil bone tissue analyses.

## 2. Results and Discussion

**Figure 1:**
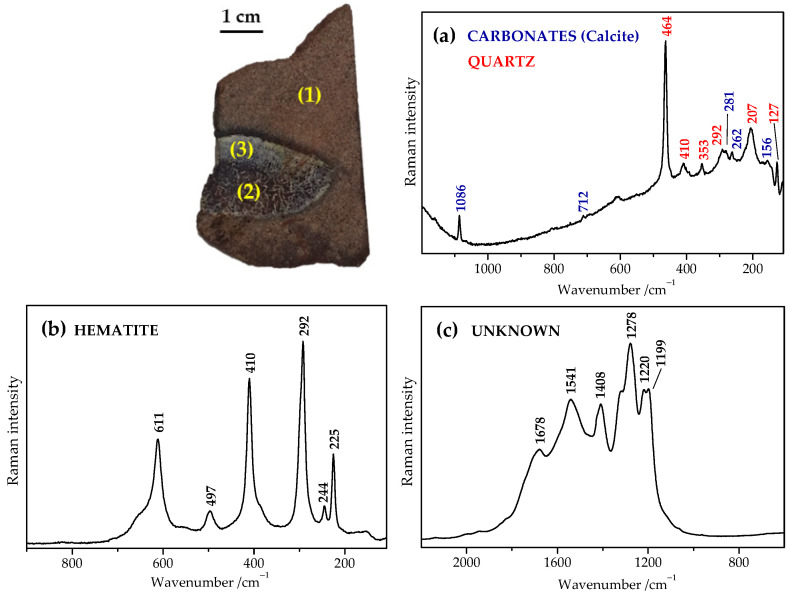
Representative Raman microspectroscopy of the fossilized bone samples (*GSIM DB2;* cross-sectional fossil reptile bone fragment collected by the Slovak-Iranian Paleontological Expedition in 2017; southern Laurasia/northern Gondwana (Iran), Mesozoic (Jurassic)). (**a**) Single-point Raman analysis of the sedimentary rock section (1) revealed common minerals including calcite and quartz. Two other Raman spectra characterized the bone thin section: (**b**) the iron oxide red pigment, hematite, was identified in the dark-red bone regions (2), and (**c**) undefined carbonate compounds were recorded in the whitish areas (3). Spectra were recorded using a 785 nm-laser excitation and are depicted directly (without any additional post-measurement processing) as an average of the spectra recorded at different points of the corresponding sections ((1) sedimentary rock; (2) and (3) bone) of the analyzed fragment.

A single-point Raman analysis was initially performed to obtain specific and structural information from the studied fossil samples. Figure 1 shows our experiment with the cross-sectional fossil reptile bone fragment. The Raman signal was collected from different surface points of the fossilized bone tissue that remained embedded in the sedimentary rock. Raman spectra recorded within the region of sediment revealed the presence of calcite and quartz (Figure 1a), which, along with gypsum, are common minerals of sedimentary rocks [85,86]. The Raman analysis of the pigmentation of fossils usually indicated the occurrence of iron oxide nanoparticles (mainly hematite (Figure 1b), but also goethite [87]); titanium dioxide crystals (anatase [88]); or manganese compounds (romanechite, pyrolusite [3,87,88,89,90,91]), which are most likely present because of the weathering, and secondary contamination processes that occurred over the diagenetic transformation. Figure 1 exhibits the case when hematite deposited inside dark-red areas of the bone section and with goethite being absent. The hematite fillings (of Haversian canals) are usually formed by the pH-dependent precipitation of ferrous hydroxide and its subsequent transformation into hematite; extensive replacement of the cortical bone matrix by hematite was demonstrated [3]. Discussion about its origin leads one to the dilemma that the bone may provide favorable sites for the iron accumulation and that this exogenous iron came from the weathered iron-bearing minerals surrounding the bone during the process of fossilization. Alternatively, we cannot rule out that an additional source of at least some iron is of endogenous (for example, hemoglobin-derived) origin. To decipher this dilemma, an in-depth analysis of well-targeted locations within the fossil bone sample must be carried out.

**Figure 2:**
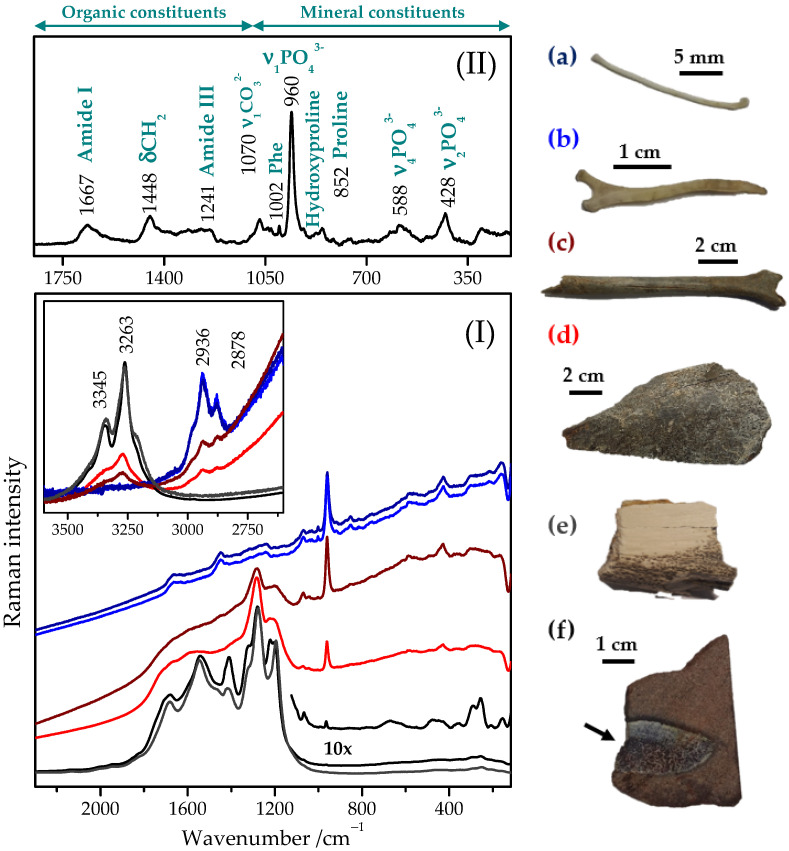
(**I**) Representative micro-Raman spectra acquired on bone fragments of *Rhynchotus* (a representative of the only extant ratites that can fly, Tinamiformes, Brazil) (**a**), and *Tinamus* (Tinamiformes, Brazil) (**b**); subfossilized bone fragments (Mammalia, non-recent Holocene, Slovakia) (**c**,**d**); *Deinocheirus* (Ornithomimosauria, Upper Cretaceous, Mongolia) (**e**); and cross-sectional bone fragment of an extinct reptile (*GSIM DB2,* Jurassic, Iran) (**f**). Spectra were acquired using a 785 nm-laser excitation. Each illustrated spectrum represents an average of the spectra recorded at different points of the compact bone selected throughout the entire bone surface; no spectral treatment (except normalization) was applied to the recorded data. Spectra were normalized for clarity of presentation to the bands at ~1280 and 430 cm^−1^. (**II**) Typical Raman spectrum of bone tissue showing the major bands and the corresponding assignments: most bands could be assigned to mineral phosphate, carbonate (below 1100 cm^−1^), or the organic phase, i.e., matrix collagen (above 1100 cm^−1^). The presented spectrum is an average spectrum of the Figure 2I(a,b) spectra. Finally, the spectrum was background corrected for clarity of presentation. (ν: stretching mode; δ: deformation-bending mode; Phe: phenylalanine).

Figure 1c shows the Raman spectrum recorded in whitish areas of the bone section. It consists of a series of very intense and broad bands localized in the 1200–1800 cm^−1^ spectral region. In particular, the bands at 1199, 1220, 1278, 1408, 1541, and 1678 cm^−1^ correspond likely to some form of the transformed carbonate compounds as we have already proposed elsewhere [92]. However, their origin is still unclear, and only some indications of their interpretation can be found in the literature [2,21,45,52,83,84,85]. The bands are localized in the spectral range characteristic of the Raman features of proteins, nucleic acids, and other important organic molecules [80,81,82,93]. Nevertheless, the recorded spectrum significantly differs when compared to the Raman spectrum of unaltered modern bone [30,31,57,58]. On the other hand, experimental outcomes exhibited similar Raman spectra despite they were collected from different Mesozoic animals of different ages and geographies. In order to enhance our interpretation of the spectra, we also analyzed bone samples of different recent and subfossil vertebrates. Some representative Raman spectra are depicted in Figure 2I. Firstly, let us look at characteristic spectral features of the bone tissue (Figure 2II).

### 2.1. Typical Raman Spectrum of Bone Tissue

Bone is a naturally occurring biphasic material comprising predominantly of a protein component (mostly collagen I) and bone mineral (biological apatite, mainly hydroxyapatite or carbonated hydroxyapatite; an inorganic calcium phosphate mineral with the stoichiometric unit cell formula Ca_10_(PO_4_)_6_OH_2_ or Ca_5_(PO_4_,CO_3_)_3_OH), as a reinforcement [57]. The primary role of biological apatite is to provide toughness and rigidity to the bone, whereas collagen provides tensile strength and flexibility [59,94]. Visually, collagen acts as a structural framework, in which plate-like tiny hydroxyapatite crystals are embedded to strengthen the bone compactness. It also contains trace ions, including carbonate, citrate, sodium, magnesium, fluoride, chloride, and potassium [53,57,60]. The minerals bind to collagen through non-collagenous proteins (~3–5 wt% of the bone), which are the active sites for biomineralization and cellular attachment [32]. Besides, other organic constituents include lipids, vascular elements, and cells, as well as water [3].

The major bands found in the Raman spectrum of bone (Figure 2II) can be assigned as follows [49]:

*(i) the organic constituents* (collagen and non-collagen moieties): Amide I at 1667 cm^−1^ (C=O stretching mode), Amide III at 1241 cm^−1^ (C-N stretching mode), C-H bending and stretching modes at 1448 and ~2800 to 3100 cm^−1^, respectively; and amino acid residues (phenylalanine at 1002 cm^−1^, proline at 852 and 918 cm^−1^, and hydroxyproline at 875 cm^−1^) visible due to the abundant type I collagen in the bone matrix [60,95] and

*(ii) the mineral constituents*: the main bands ascribed to phosphate (${\mathrm{PO}}_{4}^{3-}$) and carbonate (${\mathrm{CO}}_{3}^{2-}$) groups [96]. The Raman spectrum of bone is dominated by vibrations originating from the phosphate anion formed from one phosphorus atom bound to four oxygen atoms in a tetrahedral arrangement [53]; whereas the phosphate group has four internal vibration modes: ν_1_ (960 cm^−1^; the symmetric stretching of the phosphate moiety), ν_2_ (428/442 cm^−1^; the symmetric bend mode of ${\mathrm{PO}}_{4}^{3-}$), ν_3_ (~1075 cm^−1^; the asymmetric stretching mode of ${\mathrm{PO}}_{4}^{3-}$), and ν_4_ (588 and 620 cm^−1^; the symmetric bend mode of ${\mathrm{PO}}_{4}^{3-}$). The ν_3_ mode is usually weak in intensity and thus not seen frequently in the recorded Raman spectra [53]. Carbonate ions are also present in the bone apatite structure, where they can substitute either in the OH-site (‘A-type’ substitution) or in the PO_4_-site (‘B-type’ substitution) [33].

The internal modes of the ${\mathrm{CO}}_{3}^{2-}$ group are commonly detected at 1073 cm^−1^ and 1103 cm^−1^, corresponding to the symmetric ν_1_ stretching mode of B- and A-type carbonate, respectively [48]. However, the weakness of the A-type carbonate band usually does not permit gaining information about the composition of bone tissue; thus, only the B-type mode is commonly recognized, and up to three bands centered at 1035, 1050, and 1070 cm^−1^ can be visible [27,53,95]. Moreover, the latter tends also to be partially overlapped by a ${\mathrm{PO}}_{4}^{3-}$ band visible around 1075 cm^−1^ [30,32]. In any case, the most widely used mineral band is a phosphate band at 960 cm^−1^, which is characteristic of carbonated apatites. The exact position is sensitive to mineral carbonated and monohydrogen phosphate content [30]. In addition, the presence of a prominent carbonate band around 1070 cm^−1^ in the Raman spectrum is significant because it shows phosphate positions in the apatitic lattice susceptible to ionic substitution [32]. The peak intensity of this band was also positively correlated with the carbonate content in both the synthetic and biological samples [60]. Since carbonates have a different geometry and charge than phosphates, and as they are much bigger than hydroxyl groups, their presence in the crystal structure creates distortion that reduces the crystallinity of bioapatite compared to hydroxyapatite. Because of its low crystallinity, bone apatite is highly reactive [69,97]. It was also demonstrated that at ambient temperature, crystallinity tends to increase after death, making bioapatite less reactive. The same process also occurs during heating. Therefore, due to the higher crystallinity observed in calcined bone, the latter is more resistant to diagenesis [97], which is particularly important when studying structural modifications of fossilized bone tissue. Generally, carbonate ions found in the bioapatite crystal structure (4–7 wt%), mainly as a substitution for phosphate ions, influence the long-range atomic order of crystals and therefore play a major role in determining physical and chemical properties of bone minerals, such as solubility and crystal size [34].

### 2.2. Raman Spectra of Recent, Subfossil, and Fossil Bones

The recorded spectra of modern, subfossilized, and fossil bone fragments demonstrate strong spectral changes, especially when comparing recent and fossil specimens (Figure 2I). In particular, the Raman bands found in the spectra of the bone fragments of recent birds (Figure 2I(a,b)), which are characteristic for the bone tissue (Figure 2II), are not anymore visible in the spectra of fossil bones (Figure 2I(e,f)). On the contrary, the Raman spectra of the fossils showed new strong bands appearing above 1100 cm^−1^. In addition, the Raman spectra of recent bones are strongly affected by a relatively intense background signal, the slope of which approaches zero in the case of the fossil bones. The marked fluorescence of bone tissue, mainly assigned to its organic constituents, may significantly constrain the observation of the Raman signal. We have also experienced it despite applying the excitation with a near-infrared laser (a 785 nm diode laser), which excites little bone fluorescence [48,49,98]. Besides, bone scatters light, which also likely contributes to the observed background signal [98]. In conclusion, a certain positive tendency in the intensity of the background signal as a function of the sample fossilization stage is observed in the recorded spectra (Figure 2I): the older (recent → subfossil → fossil) the bone sample is, the lower is the fluorescent background.

Raman spectra of the subfossil bone samples (Figure 2I(c,d)) represent an intermediate stage. The bands that occurred at 1069, 960, 581, and 427 cm^−1^ are still observed, but at the same time, the organic matter bands are very weak and overlapped or even not visible. When analyzing the high wavenumbers spectral range, the bands at 2982 (*sh*), 2936, and 2878 cm^−1^ are successively replaced by those appearing at 3263 and 3345 cm^−1^ (Figure 2I inset), assuming a complete protein loss. In detail, the bands recorded in the recent bone Raman spectrum are assigned to the symmetric and asymmetric CH stretching vibration of CH_2_/CH_3_ entities of the collagen molecule. The upshifted Raman bands found in the fossil bones can be related to the stretching vibrations of hydroxyl groups characteristic for the substituted bone apatite [54,70,99,100,101]. Anyhow, all these bands can be recognized in the Raman spectra of the subfossilized bone samples. Observing such a tendency of spectral changes recorded in the Raman spectra of recent, subfossil, and fossil bone specimens, one can deduce that they could reflect a continuous and long-term fossilization process.

Thus, we assume that the recorded Raman spectra can be used as a screening tool for assessing a diagenetic stage of the analyzed (fossil) bone, where both the spectral region at higher wavenumbers and the fluorescence background signal provide indications. Obviously, a significantly higher number of bone specimens, especially subfossilized ones, need to be further analyzed to assess more accurate correlation.

Having focused on the two prominent mineral bands of the bone Raman spectrum, i.e., the phosphate and carbonate bands found at 960 and 1070 cm^−1^, respectively, we observed that the phosphate band slightly upshifted (959/960/960/962/963/965 cm^−1^) and decreased in intensity. The carbonate band shifted downwards even more slightly (1070/1070/1069/1069/1067/- cm^−1^) and disappeared when recent, subfossil, and fossil bone samples were compared to each other (Figure 2I(a–f), respectively). The fact that these prominent bands disappeared results in the impossibility of evaluating the mineral to matrix ratio, which represents a known biomarker to quantify the degree of bone mineralization [49]. Nevertheless, the ν_1_(${\mathrm{PO}}_{4}^{3-}$) band is useful to probe the atomic disorder of the apatite structure by evaluating both shifting and FWHM (Full-width at half-maximum) broadening of this band [33,53]. The ν_1_(${\mathrm{PO}}_{4}^{3-}$) position clearly demonstrates a decrease of lattice strain [29]. Further, a clear tendency of the band narrowing (almost twice; FWHM values changed from approximately 17 cm^−1^ to 9 cm^−1^ (Figure 2I(a–f)) can be deduced. Wopenka et al. [33] observed that synthetic apatite has a narrower ν_1_ band than geological apatite, which has a narrower band than bone and dentine apatite. Besides, Dal Sasso et al. [34] showed that this parameter could also be used to probe the bone crystallinity and detect diagenetically altered bone. In particular, lower FWHM values mean higher apatite crystallinity and, in the case of the modern bone, are always higher than they are for the subfossil and fossil bones [3]. Moreover, higher crystallinity is correlated to lower carbonate content. However, carbonated apatites are characteristic of an upshifted ${\mathrm{PO}}_{4}^{3-}$ ν_1_ stretching vibrational band [62]. Thus, while on the one side, we observe a loss of the carbonate content in the bioapatite chemical composition and structure, on the other hand, the presence of some carbonate-rich compounds can be deduced. Interestingly, heated apatite with carbon in its structure has been reported, with ν_1_ continuously downshifted to 943 cm^−1^ [53,67], but this is not the case of our fossil specimens.

**Figure 3:**
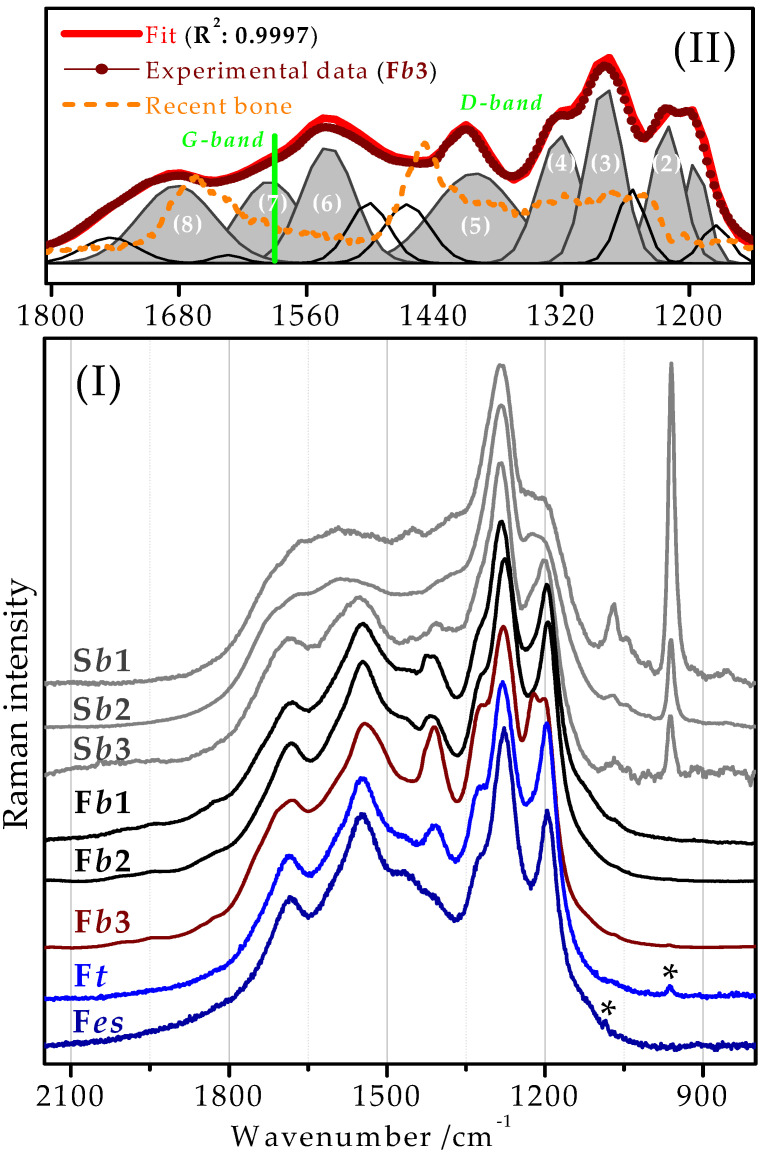
(**I**) Micro-Raman spectra of the selected subfossil and fossilized fragments of bones, an eggshell, and a tooth. For more details, see Table 1. Spectra were acquired using a 785 nm-laser excitation. All spectra were baseline-corrected and normalized to the band at ~1280 cm^−1^. Bands marked with an asterisk correspond to the phosphate and carbonate bands (F*t* and F*es*, respectively). (**II**) Curve fitting analysis of the 1150–1800 cm^−1^ Raman region characteristic for the fossilized reptile bone sample *GSIM DB2* (F*b*3). The red curve represents the resulting fitted curve displayed together with the original data (dotted-wine curve) and the Raman spectrum of the recent bone (dashed-orange curve). The peak characteristics of individual components are summarized together with tentative assignments in Table 2. The most intense/relevant peaks are highlighted and numbered. Green lines mark frequency positions of two commonly broad bands from carbonaceous material.

Finally, there are the intense unidentified (discussed) bands appearing above 1200 cm^−1^. To get more hints in their identification, we compared background subtracted Raman spectra of the subfossil and fossil bones with those of the fossil tooth and eggshell samples (Figure 3I and Table 1). In consequence, a more detailed analysis of the observed frequencies (wavenumber shifts, linewidth, relative intensities) of these bands required a deconvolution of the discussed spectral region (Figure 3II). The acquired peaks with the corresponding characteristics and tentative assignments, based on the obtained data and the bibliography [30,52,69,70,78,83,84,102,103,104], are summarized in Table 2.

Taking a closer look at the recorded spectra (Figure 3I), we could confirm previously described spectral changes. First, the Raman bands characteristic for the unaltered recent bone were no longer visible, even in the Raman spectra of the subfossil bones, or they were rare and significantly affected by the diagenesis. For instance, in the Raman spectrum of the S*b*1 sample, a weak and upshifted band of the CH_2_ bending vibrations can be seen at 1459 cm^−1^. Furthermore, the intensities of the phosphate (ν_1_$\left({\mathrm{PO}}_{4}^{3-}\right)$) and carbonate (ν_1_ (${\mathrm{CO}}_{3}^{2-}$)) bands, at ~960 and 1070 cm^−1^, respectively, progressively decreased with the advanced stage of the diagenesis until they were no longer visible in the Raman spectra of the fossilized samples. However, a very weak band at 965 and 963 cm^−1^ can be observed in the Raman spectra of the fossilized bone (F*b*3; see also the inset of the 10-times magnified spectral region of Figure 2I, black curve) and tooth (F*t*; marked with an asterisk) fragment, respectively. The upshifted position of the phosphate band indicates a probable alteration of the bone hydroxyapatite by its recrystallization. Similarly, the Raman spectrum of the fossil eggshell (F*es*) shows a very weak band at 1085 cm^−1^ (marked with an asterisk), characteristic of the calcite [85]. On the other hand, the Raman spectra of the subfossil species were defined by very intense and strongly interfered peaks, with the sharpest peak visible at ~1280 cm^−1^ (S*b*1). Apparently, these newly formed Raman bands (in comparison to the Raman spectrum of the unaltered bone tissue; Figure 2II) become better definable and increased in their intensity for the fossils. Moreover, in the case of the bone samples, they were down- or up-shifted as follows: (*i*) from 1204 to 1195 cm^−1^; (*ii*) from 1286 to 1276 cm^−1^; (*iii*) from 1406 to 1418 cm^−1^; (*iv*) from 1551 to 1546 cm^−1^; and (*v*) from 1689 to 1680 cm^−1^; whereas the average frequency shift (~9 cm^−1^) reflected relevant structural changes taking place. The F*b*3 sample was a bit peculiar, since it broke the observed tendency in some manner. Moreover, the band at ~1280 cm^−1^, visible in all spectra as a single band, was, in the case of this fossil fragment, detected as a doublet of the bands at 1197 and 1219 cm^−1^. Finally, the bands corresponding to the fossilized samples of tooth and eggshell showed agreement with the Raman spectra of the fossil bone specimens.

The principal inorganic constituents of bone and tooth are almost identical [105,106]. In fact, bones and teeth share common chemistry (collagen, hydroxyapatite, and water in various proportions), but they differ in microarchitectures and modes of growth [40]. Particularly, the tooth enamel is strikingly different. The enamel shows an extremely low porosity, a total lack of collagen (<1%), and very high mineral content. Thus, it is much more resistant to diagenetic alterations. On the contrary, dentine has similar properties as bone and is similarly susceptible to diagenesis [107]. Generally, bones and teeth share similar diagenetic histories, which are not linearly related to burial time, but rather somewhat controlled by different external factors [17].

Eggshells also have both an organic and inorganic component. They are composed of calcium carbonate (usually calcite, but also aragonite) crystals with an organic matrix of protein fibers. Their structure is preserved well deep in time, even if exposed to high temperatures. Nonetheless, diagenesis can alter their physical crystal structure and/or the chemical signature. If eggshell is composed of aragonite, then it commonly alters to calcite; if it is originally calcite, it may be more complicated, since it is not always straightforward to distinguish between primary state and secondary calcite alteration, and consequent eggshell recrystallization [108].

Having proved the discussed bands in different fossil bone samples, we assumed that these bands can be recognized as the vibrational ID profile of fossilized bone tissues. However, we cannot ignore that the same bands were also recorded in the Raman spectra of the fossilized fragments of teeth and eggshells. Therefore, they cannot be defined as a unique spectrum of fossilized bone samples. Nevertheless, it might likely be recognized as a vibrational ID profile of fossilized tissues samples.

### 2.3. Raman Mapping Images of the Fossil Bones

**Figure 4:**
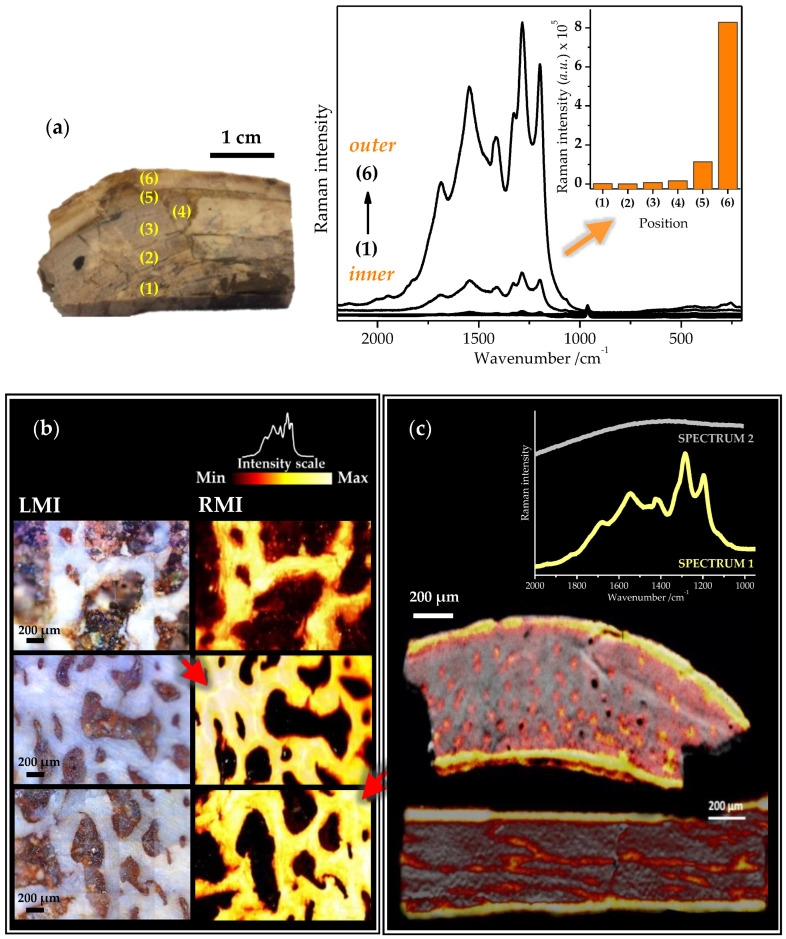
(**a**) Raman spectra of unknown carbonate compounds recorded at different positions of the fossilized tibia of *Deinocheirus mirificus.* The inset visualizes the variation of intensity of the band at ~1280 cm^−1^ as a function of the position. (**b**) Illustrative light microscopy images (LMI) and the corresponding Raman microscopy images (RMI) of the selected regions of the fossilized reptile bone sample *GSIM DB2*. Reconstructed false-color Raman mapping images show distribution of the discussed carbonate signal across the scanned areas, since the colors reflect the integrated intensities of a series of Raman bands recorded within the 1200–1800 cm^−1^ spectral range. Thus, the brightest areas correspond to the most intense Raman signal. Circumferential mineral walls surrounding the outer border of each osteon, known as the ‘cement lines’, can also be recognized (indicated by the red arrow-heads). (**c**) False-color Raman mapping images of the cross-sectional tibia fossilized bone fragments of *Yanornis* reconstructed on base of two principal spectral components: (SPECTRUM 1—yellow spectrum) the Raman spectrum related to the discussed carbonate signal and (SPECTRUM 2—grey spectrum) the fluorescence background spectrum. No more Raman spectra (except the Raman spectra of the glass microscope slide and the epoxy resin) were identified in the analyzed samples. Spectra were acquired using a 785 nm-laser excitation.

To understand more about the spatial distribution of the discussed Raman spectrum and hence about interpretation of the corresponding spectrum, we have recorded the Raman signal of the fossilized bone tissue at different positions of the bone, i.e., we have mapped the Raman signal through the section of the selected bone fragment. Figure 4a visualizes the variation of intensity of the most intense band at ~1280 cm^−1^ as a function of the position in the tibia bone sample of *Deinocheirus mirificus*. The highest Raman signal (Figure 4a, inset) was detected in the periosteal regions of the bone, i.e., the outermost part of the bone, which is closer to the surrounding sediment. In contrast, the weakest Raman signal (Figure 4a, inset) was detected in the innermost part of the bone; that is usually more protected from contact with sedimentary environment as well as factors affecting the bone during the diagenesis. Taking into account that the intensity of a Raman band is usually directly proportional to the concentration of the studied molecular system [109], we assume that the highest chemical alterations probably occurred in the outermost part of the fossilized tibia bone (Figure 4a).

Further, we have also obtained representative Raman mapping images (Figure 4b,c), allowing us to evaluate the distribution of the discussed Raman signal throughout the selected areas of the fossilized bone fragments even more complexly.

Raman mapping images obtained for the Jurassic reptile fossil bone fragment (Figure 4b) revealed an obvious detection regarding intensity of the discussed bands above at 1200 cm^−1^ in the trabecular bone. It is worthy to note that a similar microscopic appearance of the bone samples was previously associated with the late diagenetic calcite formation [3]. Furthermore, both the outermost and innermost parts of the tibia cortex of *Yanornis* showed the most intense Raman signal (Figure 4c). Besides, the transverse and longitudinal thin sections of the *Yanornis* tibia also exhibited the presence of unknown carbonates inside neurovascular canals of the bone. Thus, both peripheral and inner parts of the cortical bone were strongly altered by diagenetic processes, which ultimately led to the presumed formation of enigmatic compounds associated with the appearance of the discussed Raman bands. In conclusion, the significant impact of the surrounding environment on the chemical milieu of diagenetically altered bone may be inferred from the obtained data. 

### 2.4. Tentative Bands Interpretation

The bone fossilization is a very complex process that has been studied for centuries. Many aspects, however, are only incompletely understood. Shortly, after the death of animal, significant physical and chemical alterations of the bone constituents at different scale levels occur, promoting bioapatite recrystallization [4]. The main changes in the bone molecular structure are associated with the decay processes of organic compounds, mainly with the loss of collagen [17]. The latter varies significantly with the bone porosity, which is decisive for the formation of minerals. Hydroxyapatite of the bone remains is usually altered by recrystallization, whereas the crystal structure resulting from diagenetic alterations is thermodynamically more stable and characterized by a higher atomic order, increased crystal size, and reduced specific surface area [2,3,16,21]. The vast majority of fossilized bones exist as fluorine- and/or carbonate-enriched apatites [3,4,110,111]. 

Thus, during diagenesis, when organic remains are buried (any endogenous organic constituents are removed [20]), minerals in overlying sediments are solubilized and redeposited in pore spaces of the fossil bone. Intense chemical and isotopic exchange between the bone matrix and the environment (including percolating water) takes place, either by adsorption of ions, diffusion, ion exchange in the apatite lattice, or precipitation of secondary minerals in the pore spaces [3]. Minerals precipitated during early diagenesis occur only in those cavities in the bone accessible at that time (i.e., Haversian canals, osteocyte lacunae, and their canalicular projections). Later, they can also be precipitated in the radial cracks and along the cement lines of the secondary osteons. Common secondary mineral infillings include pyrite, calcite, quartz, manganese- or iron-(hydr)oxides, and others. Especially, secondary calcite represents a significant source of exogenous carbonate and usually occurs as a typical late diagenetic filling of Haversian canals, tectonic microcracks, and the cavities of trabecular bone [112]. Furthermore, during late diagenesis, external oxidants can also enter the fossil bone and may transform already present minerals. This is frequently the case for pyrite, which usually forms earlier and can be oxidized and transformed into hematite later on [3].

Our results conform to generally known processes that occur during diagenesis. Analyzing two prominent mineral bands of the bone Raman spectrum (phosphate (ν_1_$\left({\mathrm{PO}}_{4}^{3-}\right)$) and carbonate (ν_1_ (${\mathrm{CO}}_{3}^{2-}$)) bands, at ~960 and 1070 cm^−1^, respectively), we have observed wavenumber shifts and intensity changes. They suggest that apatite crystallinity increases with age of the bone sample. In addition, the loss of carbonate content can also be deduced. Furthermore, the Raman peak position at ca. 960 cm^−1^ for bioapatite drives towards higher wavenumbers with the addition of fluorine through diagenetic alteration [84], which is probably also the case. However, the weak bands found in the fingerprint zone (below 1000 cm^−1^) of the fossil bone Raman spectrum, namely, at 155, 257/291, 479, and 672 cm^−1^ (Figure 2I, zoomed black curve) are similar to those characteristic for the calcite and/or other carbonate minerals [85]. On the other side, the most typical band of the secondary calcite, at 1085 cm^−1^, is absent, except for the fossil eggshell (Figure 3I, F*es*). This band used to be frequently detected in fossil bones or other common exogenous carbonates [3,21]. Besides, it was also shown that carbonate concentrations in apatite of the calcined bone significantly decrease compared with a raw bone [19]. Actually, in the case of fully calcined bone, only the inorganic fraction survives, and its composition and structure are significantly modified. Moreover, the carbonate composition of archaeological samples is quite different from that of modern calcined bones [19]. The isotopic analysis demonstrated that carbonates present in the bone apatite after calcination are not all endogenous but that a significant fraction is derived from the combustion atmosphere [97]. Further, we did not find any evidence of pyrite or its associated iron oxides (except for the intense Raman bands of hematite localized if-then in totally distinct parts across the bone section; Figure 1). Finally, the recorded spectra did not show spectral characteristics common for heated or burned bone samples [19]. The presence of some kind of carbonate-rich compounds could be deduced. However, up to our knowledge, the recorded Raman bands do not agree with any Raman spectrum of common carbonates, which do not usually show high intense bands above 1000 cm^−1^ [85,113].

Recently, we have found some coincidence elsewhere [52,83,84]. Marshall et al. [52] recorded similar intense bands between ~1800 cm^−1^ and ~1000 cm^−1^ and attributed them to the presence of large-ring clusters of polycyclic aromatic hydrocarbons (PAHs). They pointed out a crucial role of the phosphate group in the formation of PAHs. In particular, they proposed that the phosphate hinders the large-scale aromatization of carbon, which tends to occur during the early stages of carbonization and gives rise to D and G bands in the Raman spectrum. Parenthetically, carbonaceous materials are characterized by the presence of the D (~1350 cm^−1^; disorder-induced band) and G band (~1590 cm^−1^; G stands for graphite) [52,114] (Figure 3II, green lines). Moreover, other similarities with our spectra could be found herein. In particular, vibrational modes of α- and γ-hematite were also detected. The γ-hematite is formed by weathering of ferrous iron and is characterized by broad and not well-defined Raman bands which are indicative of its low crystallinity. Marshall et al. [52] also observed that samples which did not preserve iron (α-hematite), contained both a weak phosphate band (at ~980 cm^−1^) as well as a series of intense bands attributed by them to PAHs compounds. Although their results were based only on a single specimen, we lean towards their interpretation of enigmatic bands. It is quite close to our assumption about the existence of some kind of carbonate-rich compounds. However, Raman spectra of PAHs themselves are subtly different [115,116]. 

Further, Yang and Wang [83] recorded Raman spectra of dinosaur specimens originating from different geographic regions and different parts of bones. Their data showed a high similarity with the spectra recorded by us, especially for the peak positions. On the other side, their spectral profiles markedly differed for different fossil specimens, which was not the case of our experimental data.

Recently, Korneisel et al. [84] presented a similar series of bands within the 1000–1700 cm^−1^ spectral range. Namely, the Raman bands at 1212, 1296, 1430, 1570, and 1690 cm^−1^ related to diagenetic kaolinite clays, since they found an agreement with photoluminescent artifact bands produced by rare earth elements (REE) in the fossil apatite and the surrounding shale matrix [117]. However, the Raman spectrum of kaolinite shows five bands in the high wavenumbers region, at 3693, 3685, 3670, 3652, and 3620 cm^−1^ [118], whose positions evidently differ when compared to our data. Regardless of that, it is worth noting that Korneisel et al. also claimed that the bone vascular canals, once purported to contain fossil red blood cells, are then filled with a mix of clay minerals and carbonaceous compounds [84].

### 2.5. Inorganic vs. Organic Origin of These Enigmatic Compounds

At the moment, we cannot determine the precise formula of the proposed carbonate-rich compounds. Indeed, further analyses have to be done and other techniques (for example, scanning electron microscopy, mass spectrometry, synchrotron-source IR spectroscopy) have to be employed to reveal more qualitative details and to describe exact mechanisms leading to their formation. Anyhow, as it was previously mentioned, we have recorded these vibrational bands in the majority of our fossil bone samples. Therefore, their exact interpretation might play an important role in the understanding of patterns and processes associated with taphonomic and diagenetic transformations of bones, especially with fossilization of biomolecules and potential preservation of biological and geological signatures in fossilized organic matter. Therefore, we would like to open a discussion regarding these enigmatic Raman bands, their origin, and assignment.

Possible preservation of biomolecules, such as proteins or DNA, is a hotly debated topic, since fossil soft tissues may provide remarkable insights into anatomy, behavior, physiology, and genomics of extinct organisms. Actually, recrystallization of the apatite matrix has the capacity to encapsulate and shelter biomolecules in newly formed crystal aggregates and thus enhance their preservation [119]. Recently, Wiemann et al. experimented with the Raman microspectroscopy detection of fossil biomolecules [9,10,75,76,77,78] and demonstrated that proteins fossilized primarily through oxidative crosslinking, whereas differences in concentrations of the proteins between tissue samples yielded different compositional trends in the resulting fossilization products [9]. In fact, the latter are products of diagenetic transformation and are preserved as carbonaceous films composed of a variety of compounds, including N- and S-heterocyclic polymers, as well as their carbonyl (C=O)- and carboxyl (COOH)-rich peroxidation products. Consequently, such fossilization products can be reliably identified via their characteristic Raman signals (C-N, C-S, and C-O vibrations), providing thus potential biomarkers for identifying the original composition of fossil soft tissues. The detailed Raman band assignments can be found elsewhere [9]. As expected, the vibrational bands described by Wiemann et al. do not agree with our measurements. However, the fossil specimens analyzed by Wiemann et al. were decalcified and mainly characterized by dark brown to gray–black coloration, which differs from those used in our analysis. Therefore, the question concerning the origin of the discussed bands (inorganic vs. organic) require numerous experiments on a much broader variety of fossilized objects.

Raman spectra of fossil bones differ significantly when compared to those of recent bones, indicating that significant chemical changes have taken place. In other words, the fossilization products exhibit a highly altered chemical and structural composition, contrasting with modern bone tissues. Although the newly formed Raman bands can be found in the spectral region representative of biomolecules, their high intensities are rather characteristic of inorganic compounds. Moreover, considering that Raman intensities tend to be directly proportional to the concentration of studied molecular system, the presence of such a high amount of organic remains cannot be assumed at all. On the contrary, such a broad and intense series of superpositioned vibrational bands are rather characteristic for both a complex system with rich intermolecular interactions (e.g., polymers) and an amorphous solid. Therefore, a possible connection with previously discussed PAHs compounds or a mixture of clay minerals and carbonaceous compounds can be found. However, we assume the formation of other minerals based on carbonate-rich compounds.

In conclusion, the same vibrational features were acquired for different fossil bones, independently of their age and depositional environments. Thus, similar spectral features could be recognized as indicators of shared traits of diagenetic history, reflecting similarities in the taphonomy of disparate fossil-bearing environments, rather than biomolecules preservation. In addition, a variety of the analyzed specimens, i.e., different fragments of fossil bones, as well as slides with thin cross-sections, implied that the recorded spectra did not coincide either with any material commonly used in a possible sample preprocessing (such as epoxy resin) or artifacts or contaminants bands [114]. Besides, a tendency observed in consecutive spectral changes (when compared to Raman spectra of recent, subfossil, and fossil bones) referred to the specificity of the analyzed vibrational bands. In addition, we demonstrated that the recorded spectra can be useful to evaluate a current diagenetic stage of the analyzed specimens. However, the same spectral features detected in the Raman spectra of the fossilized tooth and eggshell samples also indicate inorganic rather than organic residues. Finally, the acquired Raman mapping images confirmed a mineralized nature of these compounds and their probable exogenous origin. However, the fact that they were recorded exclusively in the bone (they were not seen or were seen very rarely within the sedimentary rock sections) confirmed their specificity and, at the same time, pointed out the significant effect of organic matter on their formation.

## 3. Materials and Methods

The analyzed samples were acquired as already cleaned fragments (bone, tooth, eggshell) or previously prepared thin sections of bone tissue embedded in epoxy resin. We did not perform any additional treatment on the samples. A brief summary of the analyzed samples is provided in Table 1.

All Raman spectra were obtained in the visible-near-infrared region (RL785 Renishaw diode (cw) laser; λ_exc_ = 785 nm) using a Raman confocal microspectrometer (Renishaw inVia, Great Britain) equipped with a Leica direct microscope, an electrically cooled CCD camera, and 1200 lines/mm diffraction grating. The system was calibrated and monitored using a silicon reference (520.5 cm^−1^) before the measurements. Spectra were recorded over the wavenumber range 100–4000 cm^−1^, and the sample was brought into focus using a 100× microscopic objective (NA0.9; laser spot diameter ~1 μm). The spectral resolution was 1 cm^−1^. The laser power at the sample was kept as low as possible (reasonable signal/noise ratio; no higher than 1% of the total laser power output, which represents ~2 mW) to reduce heat on samples and, thus, avoid thermal degradation. Firstly, we spectroscopically examined a possible destructive effect of the laser power applied on the sample and thus evaluated its optimal experimental value. Besides, the sample and its possible deterioration were visually checked after each measurement. The accumulation time for one spectrum was 10 s, and three accumulations were collected for a single measurement on each sample area.

In the case of the physical thin slides, we also recorded the blank Raman spectra of the glass slide, epoxy resin, and/or superglue. Possible contamination of the Raman data by these interference signals was evaluated by comparing the sample data and the corresponding blank data.

The resulting spectra were depicted directly, without any additional post-measurement processing, as an average of 10 and more spectra recorded at different areas of the corresponding specimen. The analyzed areas were chosen randomly, but systematically, in order to analyze the whole section of bone or sedimentary rock. For clarity of presentation and better spectra comparison (when indicated in the caption of the figure), Raman spectra were background-subtracted and normalized. The background was subtracted by using the Renishaw’s Windows^®^-based Raman Environment software (WiRE 3.2, Renishaw plc, Great Britain) via an “intelligent fitting” process with a polynomial value of 6. The Full-widths at half-maximum (FWHMs) of the selected bands were determined by using the SpectraGryph 1.2 optical spectroscopy software (Version 1.2.11, Menges F., Germany) [120].

Deconvolution of the 1150–1800 cm^−1^ spectral range of the Raman spectrum of the fossilized bone tissue was implemented using the Origin 9.1 program (OriginLab Corporation, Northampton, MA, USA) [121]. To obtain information regarding the number and positions of the components in the selected region for the curve-fitting process, smoothed fourth derivatives with a seven-point Adjacent Averaging function were used. This function was used instead of the second derivative function to increase the sensitivity of the peak detection [102]. The obtained band positions were used as the initial guess for the curve fitting of the original spectra. Moreover, to fit the vibrational bands, the FWHMs of the bands were constrained within reasonable limits. A Gaussian peak shape was employed, and a linear baseline was always used between 1150 and 1800 cm^−1^. Best curve fitting was obtained at the highest possible R^2^ value. Tentative assignments of the Raman band components were carried out based on the literature [30,52,69,70,78,83,84,102,103,104,115,116].

Raman imaging measurements were performed by the same Raman system (Renishaw inVia, Great Britain), using a 20× microscopic objective (NA0.4) and excitation wavelength at 785 nm. Raman spectra were collected in a StreamLine mode, which allows rapid generation of a high-definition 2D Raman images of very large sample areas. Moreover, it prevents laser-induced sample damage by illuminating with a line of laser light rather than an intense spot. The laser power used on the sample did not exceed 5% of its original power, and the exposure time was set to 4 s/spectrum. The Raman 2D reconstruction images were created by overlaying images plotted using characteristic Raman spectra recorded throughout the selected regions of the analyzed sections; by using Renishaw’s Wire 3.2 software (Renishaw plc, Great Britain) and ImageJ software (Version 1.53f51, National Institutes of Health, USA) [122]. Raman imaging results are shown in a false color representation, where the colors reflect the integrated intensities of a series of Raman bands recorded within the 1200–1800 cm^−1^ spectral range.

## 4. Conclusions

Diagenesis of fossilized bones helps to elucidate their geological history. A significant gap, however, remains in our knowledge about the fossilization process itself. Diagenetic mechanisms work in conjunction with each other, altering the biogenic composition of skeletal biomaterials. Therefore, a single experimental approach is rarely able to satisfactorily answer a diverse and multiple set of scientific issues connected with assessment of fossilized biomolecules or their residues in extinct organisms. Nevertheless, Raman microspectroscopy is the valuable analytical tool for such investigation for several reasons, including the sensitivity to minor structural changes, minimum sample preparation, high spatial resolution, and an easy and fast data acquisition.

In the present study, we applied the Raman technique to map the organic composition in a variety of fossilized organismal remains. We demonstrated that RS applied to the fossils can be used as a potential screening method to define the preservation state of sub-fossilized and fully-fossilized bones and to identify the better preserved (or less altered) parts within a sample, on which further analyses can be carried out. Both characteristic Raman bands of modern bone and diagenetically altered bone tissues were thoroughly discussed. In particular, a complex of newly appeared intense and broad bands found in the spectral region of 1000–1800 cm^−1^ were analyzed. Although these spectral features were recorded in a variety/majority of the analyzed fossil bones, no satisfactory explanation of their existence has been reached so far. The positions and spectral profiles of the bands slightly differed in a wide range of fossil bone tissues and were independent of their geographical origin. Noteworthy, the same spectral features were obtained for our fossil tooth and eggshell samples. Therefore, we suggest to further test the intense and broad bands localized in the 1200–1800 cm^−1^ spectral region as possible spectral markers for fossil tissues. The application of this criterion, together with evaluation of the fluorescence background signal and high wavenumbers bands positions, allow one to distinguish recent, subfossil, and fossil specimens. We have realized that interpretation of these bands is not so straightforward, and more experimental mapping studies and testing diverse techniques are required to assign recorded bands to specific biomolecules and specific taphonomic processes. Since our experiments do not provide any direct evidence of the possible preservation of organic remains, and the observed spectral changes reflected generally accepted processes that occur during diagenesis, the enigmatic bands were assumed to be associated with some kind of transformed carbonate-rich compounds, rather inorganic compounds than organismal residues.

The major scope of this paper was to test the feasibility of the RS approaches for biomolecule detection in the field of molecular paleontology and to provide a brief overview of related experimental achievements. Although the RS represents a powerful non-destructive, specific, and sensitive instrument, it does not automatically guarantee positive results or/and clarification of taphonomic degradation of organic residues in a variety of fossils. Further well-targeted experimental studies are required to enlarge the information database and to elaborate our empiric experience and understanding spectral specifics of the Raman response to chemical and mineralogical changes due to variable fossilization factors.

## Figures and Tables

**Table 1:** Summary of the analyzed subfossil and fossilized bone, tooth, and eggshell samples, and those spectra are depicted in Figure 3I.

Sample			Origin		Figure
			Time	Place	
**Subfossil** **bone**	**S*b*1**	mammal	Holocene	Slovakia	Figure 2I(c)
	**S*b*2**	mammal	Holocene	Slovakia	Figure 2I(d)
	**S*b*2**	*Aepyornis* (ratite bird)	~1000 AD	Madagascar	---
**Fossil** **bone**	**F*b*1**	*Yanornis* (basal bird)	Mesozoic (Early Cretaceous)	Liaoning (China)	Figure 4c
	**F*b*2**	*Deinocheirus* (ornithomimosaur dinosaur)	Mesozoic (Upper Cretaceous)	Gobi (Mongolia)	Figure 2I(e) and Figure 4a
	**F*b*3**	*GSIM DB2* (reptile)	Mesozoic (Jurassic)	Kerman (Iran)	Figure 1, Figure 2I(f) and Figure 4b
**Fossil** **tooth**	**F*t***	ceratopsian dinosaur	Mesozoic (Lower Cretaceous)	Kemerovo (Russia)	---
**Fossil** **eggshell**	**F*es***	dinosaur	Mesozoic (Upper Cretaceous)	Inner Mongolia (China)	---

**Table 2:** Recorded Raman frequencies resulted from the curve fitting analysis (Figure 3II) of the discussed spectral region and some of the corresponding tentative assignments. The most intense/relevant frequencies are highlighted and numbered. The Raman shifts observed in the non-treated spectra (Figure 3I) are also present (where applicable).

Raman ^a^/cm^−1^	Peak Numbering and the Evidenced Frequency Shifts		Tentative Vibrational Assignment ^b^
1175 *wm*			ν(CC), ν(COC), ν(CN)
**1197 *ms***	**(1)**	/1204 **←** 1195 cm^−1^	δ(CCH), δ(COC)
**1219 *s***	**(2)**		ν(CO), δ(CH_2_), δ(CH_3_), δ(NH_2_)
1253 *m*			ν(CO), δ(CC)
**1281 *vs***	**(3)**/1294 **←** 1276 cm^−1^		ν(CC), ν(CO), δ(CC)
**1320 *s***	**(4)**/1320 ↔ - cm^−1^		δ(CH), δ(CN)
**1399 *ms*, *br***	**(5)**/1406 **→** 1418 cm^−1^		$\delta ({\mathrm{CH}}_{2}),\;\delta ({\mathrm{CH}}_{3}),\;\nu_{s}({\mathrm{CO}}_{2}^{-}$)
1466 *m*			δ(OH), δ(CH_2_), δ_as_(CH_3_)
1499 *m*			
**1539 *ms***	**(6)**/1551 **←** 1546 cm^−1^		δ_s_(NH), δ(CN)
**1595 *m*, *br***	**(7)**/-------- - ----------		ν(C=C)
1634 *vw*			$\nu (\text{-}\mathrm{COOH})/\nu_{\mathrm{as}}({\mathrm{CO}}_{2}^{-}$)
**1685 *m*, *br***	**(8)**/1689 **←** 1680 cm^−1^		$\nu (\text{-}\mathrm{COOH})/\nu_{s}({\mathrm{CO}}_{2}^{-}$)
1746 *wm*, *br*			ν(C=O)

^a^ *vw*, very weak; *w*, weak; *wm*, weak medium; *m*, medium; *ms*, medium strong; *s*, strong; *vs*, very strong; *br*, broad; ^b^ ν, stretching; δ, bending/deformation; as—asymmetric; s—symmetric.

## Data Availability

Not applicable.
